# Increasing Prevalence of Nontuberculous Mycobacteria in Respiratory Specimens from US-Affiliated Pacific Island Jurisdictions^1^

**DOI:** 10.3201/eid2403.171301

**Published:** 2018-03

**Authors:** Chunrong Lin, Chad Russell, Bruce Soll, Dominic Chow, Sapna Bamrah, Richard Brostrom, Wesley Kim, Jerry Scott, Matthew J. Bankowski

**Affiliations:** University of Hawaii at Manoa, Honolulu, Hawaii, USA (C. Lin, C. Russell, B. Soll, D. Chow, W. Kim, J. Scott, M.J. Bankowski);; Centers for Disease Control and Prevention, Atlanta, Georgia, USA (S. Bamrah, R. Brostrom);; Diagnostic Laboratory Services, Inc., Aiea, Hawaii, USA (W. Kim, M.J. Bankowski);; Queen’s Medical Center, Honolulu (W. Kim, M.J. Bankowski)

**Keywords:** Mycobacterium tuberculosis, MTB, bacteria, tuberculosis and other mycobacteria, nontuberculous mycobacteria, NTM, Mycobacterium avium complex, MAC, epidemiology, prevalence, respiratory specimens, respiratory infections, US-affiliated Pacific Island jurisdictions

## Abstract

Nontuberculous mycobacteria (NTM) respiratory infections represent a growing public health problem in many countries. However, there are limited published epidemiologic studies for the Western Pacific region. We reviewed respiratory specimens submitted to Diagnostic Laboratory Services in Hawaii, USA, for culture of *Mycobacterium tuberculosis* during August 2007–December 2011 to determine the NTM isolation rate. We observed a statistically significant increase in the rate of specimens with NTM isolated in respiratory culture (adjusted rate ratio per year 1.65, 95% CI 1.54–1.77; p<0.01). In contrast, the number of patients with respiratory cultures positive for *M. tuberculosis* showed no increase (adjusted rate ratio per year 0.98, 95% CI 0.94–1.01; p = 0.19). A 6-month subset of NTM isolates was identified by using a nucleic acid probe or 16S rRNA sequencing. *M. avium* complex and *M. fortuitum* were the most common NTM identified.

Nontuberculous mycobacteria (NTM) are ubiquitous in the environment and have been identified repeatedly from the soil and municipal and other water supplies by using both molecular and traditional methods (*1*–*3*). NTM might vary in their pathogenicity, but are most commonly associated with pulmonary infections (*4*). The incidence of NTM pulmonary infections is increasing and is an emerging public health concern (*5*–*8*). It has been proposed that the increased use of piped, chlorinated water might serve as a common thread between the increasing incidence of NTM pulmonary disease in the industrialized and developing worlds, especially those infections caused by the *Mycobacterium avium* complex (MAC) (*9*). The resistance of NTM to chlorine might enable NTM to have a selective advantage in their microenvironment (*2*). NTM have also been isolated from shower heads, which provide a moist and conducive environment for NTM to grow and form biofilms. Consequently, NTM might be aerosolized and then inhaled (*10*), thereby causing pulmonary infection and disease in susceptible persons. Use of more accurate detection methods has also contributed to a documented increase in NTM-associated disease (*6*).

The incidence and prevalence of NTM and associated respiratory disease is unknown for US-affiliated Pacific Islands (USAPI). The specific islands or atoll chains involved in this study are composed of 6 jurisdictions that have formal relationships with the United States: the territories of American Samoa and Guam, the Commonwealth of the Northern Mariana Islands, and the Freely Associated States. These states are the Republic of Palau, the Republic of the Marshall Islands, and the Federated States of Micronesia (the island states of Chuuk, Kosrae, Pohnpei, and Yap) (*11*). The United States and each of the Freely Associated States have a Compact of Free Association that provides citizens of these nations the rights of entry, residence, and employment without being subjected to the usual health screening required for new immigrants from other countries (*12*).

The Centers for Disease Control and Prevention (CDC) has been actively involved in the prevention and control of several infectious diseases, including tuberculosis, in the USAPI. In 2010, the USAPI regional rate of infection with *M. tuberculosis* (MTB) (106.0 cases/100,000 persons) was >12 times the rate in Hawaii (8.8 cases/100,000 persons) and almost 30 times the US national rate (3.6 cases/100,000 persons) (*13*). In contrast, there is no systematic monitoring of NTM-related respiratory infections, and thus there is no information on the epidemiology and potential role of NTM in respiratory disease in the USAPI.

Reports from other regions indicate that the epidemiology of NTM can be highly variable (*6*). Patients with previous pulmonary MTB infections are also at an increased risk for development of pulmonary NTM infections. This finding might be caused by structural lung damage arising from pulmonary MTB or because of host defense or immune defects (*4*).

We conducted this study to investigate the prevalence of NTM isolation in respiratory specimens from the USAPI. Description of the prevalence of NTM pulmonary isolates and disease in the USAPI can have major implications in screening MTB disease and NTM disease in this region and in regions to which patients immigrate.

## Methods

### Specimen Collection

Diagnostic Laboratory Services (DLS) (Aiea, HI, USA) performed specimen testing for acid-fast bacilli (AFB) under the scope of a federal contract; Guam was not included in this contract during the years studied and is not included in this analysis. The primary focus of the contract was on enabling effective MTB control through the delivery of laboratory and diagnostic services to the USAPI. Respiratory specimens consisting of either 2 or 3 expectorated sputum samples were collected within a 24-hour period according to recommendations of the World Health Organization or CDC. Only MTB complex was definitively identified; all other *Mycobacterium* spp. were reported to the respective USAPI as NTM without further identification. Samples were obtained primarily from patients undergoing evaluation for possible MTB disease either because of tuberculosis symptoms or from close contact with patients with infectious MTB disease.

### Laboratory Testing

Specimens were digested by using the standard NALC-NaOH method, followed by centrifugation (*14*). A portion of the sediment was then used to prepare an AFB smear. The remaining sediment was resuspended and used to inoculate 7H9 liquid medium (BACTEC MGIT 960; Becton Dickinson, Franklin Lakes, NJ, USA), broth medium, and solid medium (Middlebrook 7H10 agar slant; Becton Dickinson). The broth medium was incubated in a MGIT 960 semiautomated system (Becton Dickinson) and the 7H10 agar slant was incubated at 35°C in an incubator containing 5% CO_2_. The broth was monitored for mycobacterial growth for <6 weeks, and the 7H10 agar slant was monitored for 8 weeks.

The prepared smear was heat-fixed at 70°C–80°C for 2 h. The smear was then stained by using the fluorochrome stain method, which used auramine O, a decolorizing solution, and a potassium permanganate counterstain and read by using a fluorescent microscope. All AFB smear-positive specimens were analyzed by using the Gen-Probe Amplified Mycobacterium Direct Test (Hologic, Marlborough, MA, USA) for nucleic acid amplification testing specific for the MTB complex.

All AFB-positive cultures were also tested for MTB by using the MTB AccuProbe System (Hologic), which is used to directly identify the mycobacterial species in MTB-positive cultures (i.e., *M. tuberculosis*, *M. bovis*, *M. africanum*, and *M. microti*). If the AFB-positive culture was negative for MTB by MTB AccuProbe, the isolate was routinely reported only as an NTM without speciation according to the CDC contract.

### Subset Analysis

All consecutive NTM-positive respiratory specimens collected during May–November 2010 were further analyzed to identify the species of NTM from the region. A total of 35 respiratory specimens, all from different patients, were positive for NTM during that period. Samples exhibiting features of MAC by light microscopy were initially tested by using the AccuProbe *Mycobacterium avium* Complex Test (Hologic). This nucleic acid test identifies mycobacterial species in the MAC (e.g., *M. avium*, *M. intracellulare*, and others). Specimens with negative results for MAC by AccuProbe were then further identified by using 16S rRNA sequencing (MicroSeq 500-bp 16S rDNA and MicroSeq ID Analysis software; Thermo Fisher Scientific, Waltham, MA, USA) as described by Hall et al. (*15*). NTM identification by 16S rRNA sequencing was analyzed by using RipSeq software (Pathogenomix, Santa Cruz, CA, USA) and a corresponding database (*16*).

Epidemiologic and clinical data were obtained on the described subset of patients by paper chart review at respective MTB treatment centers. Data were successfully obtained for 29 (82.9%) of 35 patients. This information was relayed back to DLS in a secure manner and deidentified before analysis. This study was approved by the institutional review board of the Queen’s Medical Center and each of the involved USAPI jurisdictions.

### Statistical Analysis

Descriptive statistics included frequencies and prevalence percentages calculated by participant characteristics and calendar year. The outcome variable was a positive mycobacteria culture. Mycobacteria cultures were further defined as NTM or MTB. Patients with multiple positive cultures of the same mycobacterial species (i.e., MTB or NTM) were counted only once. Patients who were subsequently positive for a different species of mycobacteria (e.g., positive for MTB in 2008 and NTM in 2010) were counted as having 2 separate cases. Variables of interest were calendar year, age, sex, and geographic site. We calculated annual and overall period prevalence for each case group by using population data obtained from the US Census Bureau. We used Poisson regression models to estimate rate ratios of positive mycobacteria cultures per year for NTM and MTB. We further adjusted all estimates by age group, sex, and geographic site to determine the relative prevalence of positive cultures. Results of the regression models are expressed as relative rates with 95% CIs. Analyses included unadjusted models and models adjusted for age or for age, sex, and geographic sites. A p value <0.05 was considered statistically significant. We performed analyses by using SPSS software (IBM Corporation, Armonk, NY, USA) and SAS software (SAS Institute, Cary, NC, USA).

## Results

A total of 15,811 respiratory specimens collected from 5,807 patients were sent to DLS for AFB smear and culture during August 2007–December 2011. A total of 998 patients had >1 AFB-positive respiratory culture: 675 (67.6%) were MTB positive and 323 (32.4%) were NTM positive. Five patients were classified as being co-infected.

We obtained baseline characteristics for patients from the various regions of the USAPI (Table 1). Patients with NTM respiratory isolates were older than the overall USAPI population (median age 34.8 years vs. 24.0 years), but a similar proportion were female (49.2% vs. 49.1%). MTB-positive patients were also older than NTM-positive patients (median age 42.6 years vs. 34.8 years); however, sex was similar (49.3% female vs. 49.2% male).

**Table 1:** Baseline characteristics of patients from various regions in the US-Affiliated Pacific Islands with respiratory tract culture specimens tested for AFB, August 2007–December 2011*

Characteristic	Total population†	AFB positive		AFB negative
		NTM	MTB	
Total	304,542 (100)	323 (0.1)	675 (0.2)	4,804 (2)
Median age, y	24.0	34.8	42.6	39.9
Sex, %				
M	50.9	50.8	50.7	54.4
F	49.1	49.2	49.3	45.6
Island nations				
American Samoa	55,529 (18.2)	12 (3.7)	10 (1.4)	120 (2.5)
Commonwealth of the Northern Mariana Islands	55,121 (18.2)	29 (9.0)	87 (12.8)	522 (11.5)
Federated States of Micronesia	107,154 (35.1)	176 (54.5)	266 (39.4)	2,140 (44.5)
Palau	20,879 (6.8)	32 (9.9)	51 (7.5)	882 (18.4)
Republic of the Marshall Islands	65,859 (21.6)	74 (22.9)	261 (38.6)	1,140 (23.7)

*Values are no. (%) patients unless otherwise indicated. American Samoa, Federated States of Micronesia, and the Commonwealth of the Northern Mariana Islands reported 2, 2, and 1 patients with co-infections, respectively. Specimens from Guam were not included in specimens provided to the Diagnostic Laboratory Services during this period. AFB, acid-fast bacilli; MTB, *Mycobacterium tuberculosis*; NTM, nontuberculous mycobacteria. †Data were obtained from https://www.census.gov/population/international/data/idb/informationGateway.php.

The rate of NTM isolation increased from 0.5% of patients screened in 2007 to 11.3% of patients screened in 2011. Conversely, the rate of MTB detection in the population screened remained relatively stable but showed a statistically significant change from 11.5% in 2007 to 13.7% in 2008 (p = 0.001) (Table 2). However, there was no major change thereafter.

**Table 2:** Age- and site-adjusted relative rates of positive cultures by year, US-affiliated Pacific Islands, August 2007–December 2011

Bacteria and year	No. patients screened	Positive cultures, %	Adjusted relative rates (95% CI)*	p value
Nontuberculous mycobacteria				
2007†	595	0.53	1 (referent)	
2008	996	3.31	7.53 (3.20–17.71)	<0.001
2009	1,224	5.99	14.22 (6.24–32.36)	<0.001
2010	1,556	6.37	19.48 (8.62–44.05)	<0.001
2011	1,436	11.32	29.04 (12.89–65.41)	<0.001
*Mycobacterium tuberculosis*				
2007†	595	11.54	1 (referent)	
2008	996	13.71	1.40 (1.15–1.69)	0.001
2009	1,224	12.41	1.14 (0.95–1.36)	0.17
2010	1,556	12.36	1.05 (0.88–1.25)	0.60
2011	1,436	11.67	1.07 (0.89–1.28)	0.45

*Adjusted for age and geographic sites. †August–December 2007.

We observed an increasing trend in annual prevalence of patients who were NTM culture positive from August 2007 (i.e., extrapolated for 2007) through December 2011 (Figure). This prevalence increased from 2 cases/100,000 persons in 2007 to 48 cases/100,000 persons in 2011 (adjusted rate ratio 1.65, 95% CI 1.54–1.77; p<0.01). In contrast, annual prevalence of MTB remained relatively stable over the same period and fluctuated from 43 cases/100,000 persons in 2008 to 58 cases/100,000 population in 2010 (adjusted rate ratio 0.98, 95% CI 0.94–1.01; p = 0.19) (Table 3).

**Figure:**
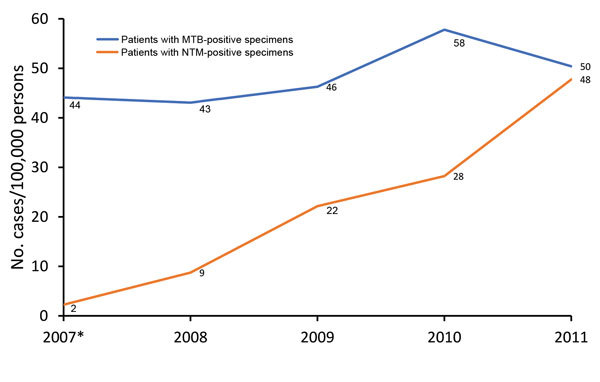
Prevalence of positive test results for NTM and MTB in respiratory specimens from patients in US-affiliated Pacific Island jurisdictions, 2007–2011. *Data for 2007 were extrapolated from data for August–December 2007. MTB, *Mycobacterium tuberculosis*; NTM, nontuberculous mycobacteria.

**Table 3:** Rate ratios for positive mycobacteria cultures per year in US-affiliated Pacific Islands, August 2007–December 2011

Variable	Unadjusted		Adjusted*	
	Rate ratio per year (95% CI)	p value	Rate ratio per year (95% CI)	p value
Negative culture	1 (referent)		1 (referent)	
*Mycobacterium tuberculosis*	0.98 (0.93–1.04)	0.55	0.98 (0.94–1.01)	0.19
Nontuberculous mycobacteria	1.57 (1.43–1.74)	<0.001	1.65 (1.54–1.77)	<0.01

*Adjusted for age, sex, and site.

The overall period prevalence of NTM isolation for our study period (August 2007–December 2011) was 106 cases/100,000 persons. Age data were available for 244 (75.5%) of 323 patients with NTM-positive specimens. Prevalence for persons 50–64 years of age (242 cases/100,000 persons) and for persons >64 years of age (278 cases/100,000 persons) wereas more than double the overall prevalence. Prevalence of NTM isolation varied greatly for USAPI regions; the lowest rate was observed in American Samoa (22 cases/100,000 persons) and the highest rate was observed in the Federated States of Micronesia (164 cases/100,000 persons).

The cumulative rate for MTB isolation over the same period was 221 cases/100,000 persons. This rate was highest for persons 50–64 years of age (330 cases/100,000 persons) and similar for persons 15–49 years of age (229 cases/100,000 persons) and persons >64 years of age (211 cases/100,000 persons). The highest rate was observed in the Republic of the Marshall Islands (396 cases/100,000 persons), more than 20-fold higher than the rate in American Samoa (18 cases/100,000 persons). We also determined period prevalence for the rest of the island nations and stratified prevalence by age groups (Table 4).

**Table 4:** Period prevalence of persons with >1 AFB-positive culture in US–affiliated Pacific Islands, August 2007–December 2011*

Characteristic	Total population	NTM	MTB
Territory			
American Samoa	55,529	12 (22)	10 (18)
Commonwealth of the Northern Mariana Islands	55,121	29 (53)	87 (158)
Federated States of Micronesia	107,154	176 (164)	248 (266)
Palau	20,879	32 (153)	51 (244)
Republic of the Marshall Islands	65,859	74 (112)	261 (396)
Total no. cases	304,542	323 (106)	675 (221)
Cases with age data, no. (%)	NA	244 (75.5)	541 (80.1)
Age, y			
0–14	101,812	31 (30)	44 (43)
15–49	158,365	102 (64)	363 (229)
50–64	33,932	82 (242)	112 (330)
>64	10,433	29 (278)	22 (211)

*Values are no. with positive specimens (rate/100,000 persons) except as indicated. Population data were obtained from the US Census Bureau (http://www.census.gov/population/international/index.html) by using the midyear population in 2010. MTB, *Mycobacterium tuberculosis*; NA, not applicable; NTM, nontuberculous mycobacteria.

### Subset Analysis with NTM Identification

A total of 35 consecutive patients who had respiratory specimens were positive for NTM during May 31–November 12, 2010. We further identified specimens to the species level by using AccuProbe MAC or 16S rRNA sequencing. We conducted database comparison by using GenBank or RipSeq (Pathogenomix). The mycobacterial species identified in these respiratory specimens were 11 (31.4%) MAC (including *M. intracellulare* and *M. chimaera*), 7 (20.0%) *M. fortuitum*, 5 (14.3%) *M. gordonae*, 2 (5.7%) *M. abscessus/chelonae*, 2 (5.7%) *M. parascrofulaceum/M. fortuitum*, 1 (2.9%) *M. kansasii*, 1 (2.9%) *M. florentinum*, 1 (2.9%) *M. mucogenicum*, 1 (2.9%) *M. paraffinicum*, 1 (2.9%) *M. simiae*, and 1 (2.9%) *M. terrae*. Two specimens were positive for >1 NTM species: 1 (2.9%) *M. fortuitum* and *M. triplex*, and 1 (2.9%) *M. fortuitum* and *M. gordonae*.

By paper chart review, we obtained clinical data for 29 of 35 patients with sputum samples positive for NTM during May 12–November 12, 2010. Median age for these patients was 51 years (range 8–86 years). Nineteen (65.5%) of 29 responders were male, median height was 163 cm, and median weight was 59 kg. Most patients were unemployed, retired, or did not provide any information on their occupation. Self-reported ethnicity was 86.2% (25/29) Pacific Islanders, 6.9% (2/29) Filipinos, 3.4% (1/29) Chinese, and 3.4% (1/29) unknown.

Chronic cough, sputum production, weight loss, and fatigue/malaise were the most commonly reported signs or symptoms. The most common concurrent diagnosis involving the lung was chronic obstructive pulmonary disease, which affected 8 (27.6%) of 29 patients for whom clinical data were collected. A total of 6 (31.0%) of 29 patients were current smokers, and 3 (10.3%) of 29 had been given a diagnosis of diabetes mellitus. There was a paucity of radiologic investigations available for USAPI patients; only 2 patients had chest radiographs performed. Radiographic findings showed nodular lesions and 1 cavitary lesion (*1*) and multiple cavitary lesions with bronchiectasis (*1*). Thirteen of the 29 patients had received empiric therapy for MTB.

## Discussion

Data for this study identified major epidemiologic trends and differences in the prevalence of NTM isolation in the USAPI. We found a major increase in annual prevalence of patients with NTM respiratory isolates in the USAPI during August 2007–December 2011. The annual prevalence of NTM isolation in 2011 (48 cases/100,000 persons) is similar to recently published data for Hawaii (*8*).

Increased testing, as shown by gradually increased number of patients screened, probably contributed to the upward trend in NTM isolation. However, the rate of increase of NTM isolation seems out of proportion to the modest increase in the number of patients screened. It is possible that actual prevalence of NTM respiratory diseases is increasing in this region in a manner similar to that of neighboring Hawaii (*8*) because most patients had respiratory signs and symptoms and were suspected to have MTB infections. Our findings are also consistent with those reported for the continental United States, where the prevalence of NTM pulmonary isolates has been shown to be increasing (*6*,*7*).

The high prevalence of NTM infections in the USAPI might also be attributable to environmental conditions in the area. Adjemian et al. postulated that soil composition and greater persistence of moisture droplets in the air contributed to the increased prevalence of NTM infection in Hawaii (*8*). It is likely that similar environmental conditions are present in the USAPI, although specific and systematic environmental sampling studies will need to be conducted to confirm this possibility.

The increase in NTM infections in other countries has been associated with urbanization and increased availability of piped water. The urban population growth rate during 2010–2015 of the various study regions was –0.1% for American Samoa, 0.3% for the Federated States of Micronesia, 0.4% for the Commonwealth of the Northern Mariana Islands, 0.6% for the Republic of the Marshall Islands, and 1.7% for Palau (*17*). Although information about access to piped water for our patients is not available, the stable urban/rural makeup of the population in these regions makes access to piped water an unlikely cause for the increased prevalence of NTM.

The prevalence of NTM isolation showed major variations for different island nations. Although the urbanization rate did not change much over our collection period, the degree of urbanization within the different nations might be contributing to differences in prevalence for the nations. The Federated States of Micronesia, which had the highest period prevalence of NTM isolation, also had the lowest percentage of its population living in urban areas (22.4%) (*17*). Conversely, American Samoa, which had the lowest prevalence of NTM isolation, also had a relatively high urban population (87.2%). Variations in the percentage of urban dwellers in the other regions were less drastic: 72.7% for the Republic of the Marshall Islands, 87.1% for Palau, and 89.2% for the Commonwealth of the Northern Mariana Islands. More studies are needed to evaluate the relative contribution of each factor to the rate of NTM isolation and disease. 

The highest NTM isolate rates were observed for older persons (those 50–64 years of age and those >64 years of age). This finding is consistent with results from another study (*6*).

NTM identification in the limited subset analysis showed numerous NTM with variable pathogenicity. Although MAC was the most commonly isolated NTM species in our study, its isolation was not as common in the USAPI as it was in North America and Europe (*4*,*18*). Rapidly growing mycobacteria, in particular *M. fortuitum*, appear to be comparatively more prevalent in the subset. This finding is supported by epidemiologic studies in Asia, which suggested that Pacific Islanders, similar to Asians, might have an ethnic susceptibility to rapidly growing mycobacteria. Subset analysis in our study suggests that the predominant NTM species in the USAPI might be similar to the NTM species reported from Asia (*18*).

Almost half of the subset patients surveyed had evidence of treatment for infection with MTB. It is common to start effective MTB therapy in patients with a high pretest probability of MTB. However, only 2 of these patients had chest radiographs performed. This finding is a reflection of limited resources available in clinics in this region. In these patients, routine use of direct nucleic acid amplification testing (e.g., GeneXpert MTB/RIF; Cepheid Inc., Sunnyvale, CA, USA) for AFB-positive smears might provide more support for final diagnosis and treatment options.

In contrast to findings for NTM, the prevalence of patients with MTB-positive respiratory cultures remained relatively stable over the study period. The Marshall Islands have been identified as having the highest rate of tuberculosis infections in the Western Pacific islands (and one of the highest rates worldwide); this rate has been linked to high rates of diabetes in the region (*19*). The epidemiology of MTB in the region has been extensively discussed (*13*,*20*,*21*).

This study had several limitations. First, the study used microbiological data for patients who were suspected to have MTB infection and thus might not be representative of the entire population. However, the most common signs and symptoms of NTM infection (chronic or recurring cough, fatigue, malaise, dyspnea, fever, hemoptysis, chest pain, and weight loss) are not dissimilar from those with MTB infection (*4*). Furthermore, because testing was not performed primarily to detect NTM infection, it is more likely that our study underestimated the prevalence of NTM isolation, and that the actual prevalence is probably higher. Second, although the number of patients from whom respiratory samples were collected remained relatively stable, the total number of respiratory specimens submitted increased over time. This limitation might have contributed to the increasing trend of NTM-positive respiratory specimens in our data. Third, data collection systems in the region were rudimentary, and only sex and age data were provided to DLS during specimen collection. Fourth, identification of NTM-positive respiratory specimens was not routinely performed, and nonpathogenic species, such as *M. gordonae*, were not excluded, potentially overestimating the prevalence of NTM respiratory infections in the region.

Identification data were obtained for a limited subset of 35 patients, and further studies should be conducted to identify the composition of subspecies from the region. Clinical and epidemiologic data for this group are incomplete and cannot be used to make meaningful conclusions about the rest of the study population.

In conclusion, the prevalence of patients with NTM respiratory isolates from the USAPI is higher than that reported in data for the continental United States; this rate steadily increased during 2007–2011. Although these data were obtained from a cohort suspected to have MTB infection and might not be representative of the general population, the high proportion of patients with suspected MTB that cultured NTM has major clinical and public health implications. A limited subset analysis in this study suggested that there might be relatively fewer MAC isolates and a greater number of *M. fortuitum* isolates in the USAPI than in North America and Europe. Further studies and more data are required to increase our understanding of NTM infection in this region.
